# Isolation mediates persistent founder effects on zooplankton colonisation in new temporary ponds

**DOI:** 10.1038/srep43983

**Published:** 2017-03-09

**Authors:** Anna Badosa, Dagmar Frisch, Andy J. Green, Ciro Rico, Africa Gómez

**Affiliations:** 1 Department of Wetland Ecology, Estación Biológica de Doñana (EBD-CSIC), c/Américo Vespucio s/n, 41092 Sevilla, Spain; 2 Aquatic Ecology Group, University of Vic - Central University of Catalonia, c/ de la Laura, 13, 08500 Vic, Spain; 3 School of Environmental Sciences, University of Hull, HU6 7RX, United Kingdom; 4 School of Biosciences, University of Birmingham, B15 2TT, United Kingdom; 5 School of Marine Studies, Molecular Diagnostics Laboratory, The University of the South Pacific, Laucala Campus, Suva, Fiji

## Abstract

Understanding the colonisation process in zooplankton is crucial for successful restoration of aquatic ecosystems. Here, we analyzed the clonal and genetic structure of the cyclical parthenogenetic rotifer *Brachionus plicatilis* by following populations established in new temporary ponds during the first three hydroperiods. Rotifer populations established rapidly after first flooding, although colonisation was ongoing throughout the study. Multilocus genotypes from 7 microsatellite loci suggested that most populations (10 of 14) were founded by few clones. The exception was one of the four populations that persisted throughout the studied hydroperiods, where high genetic diversity in the first hydroperiod suggested colonisation from a historical egg bank, and no increase in allelic diversity was detected with time. In contrast, in another of these four populations, we observed a progressive increase of allelic diversity. This population became less differentiated from the other populations suggesting effective gene flow soon after its foundation. Allelic diversity and richness remained low in the remaining two, more isolated, populations, suggesting little gene flow. Our results highlight the complexity of colonisation dynamics, with evidence for persistent founder effects in some ponds, but not in others, and with early immigration both from external source populations, and from residual, historical diapausing egg banks.

Understanding natural colonisation processes is crucial to the interpretation of species diversity patterns^1^, the prediction of the spread of alien species^2^,^3^,^4^ and the management of habitat restoration and community reestablishment. The interplay of evolutionary forces is especially intense during colonisation, with long lasting effects on the genetic structure of populations^5^,^6^. In continental aquatic habitats, zooplankton display high dispersal and colonisation abilities^7^,^8^,^9^, but genetic differentiation can be extensive even between neighbouring populations^10^,^11^. Persistent founder effects, resulting from colonisation from a few individuals and subsequent rapid growth rates and therefore a numerical advantage of the first colonists^11^,^12^,^13^, rapid genetic adaptation to local conditions^14^,^15^, and the build-up of large dormant egg banks^16^, can lead to monopolization of resources by genotypes descending from the initial founders^10^. De Meester *et al*.^10^ summarised these processes as the ‘Monopolization hypothesis’, which is especially relevant for cyclically parthenogenetic zooplankters, such as monogonont rotifers and cladocerans. In populations of the rotifer *Brachionus* and the waterflea *Daphnia*, persistent founder events result in low levels of gene flow between nearby ponds and in high genetic differentiation^11^,^17^. Several studies have emphasized the key role of monopolization in shaping the genetic structure of populations^15^,^18^.

However, it is currently unknown at what stage during the colonisation process monopolisation will become apparent. During the early stages of population establishment, monopolization by the first colonists may be counteracted by other factors favouring ongoing migration^10^. For example, newly established dormant egg banks may be relatively small and lack ecologically relevant variation, reducing their buffering role against immigrant genes 19,20. Inbreeding depression is also likely to occur, since colonising populations often descend from a few founder genotypes which will likely result in high selfing rates^18^. Additionally, in cyclical parthenogens, clonal selection throughout the growing season (when reproduction is parthenogenetic) could reduce the number of genotypes available for sexual reproduction, resulting in a low effective population size^13^,^21^. As a consequence, later immigrants with a strong fitness advantage could hybridize with inbred residents (outbreeding), making gene flow more effective and reducing monopolization effects. This has been shown for young *Daphnia* metapopulations, where outbred genotypes rapidly increase their frequency due to hybrid vigour (competitive advantage), leading to the incorporation of new alleles in subsequent generations^19^,^22^,^23^. Given the inbreeding depression and hybrid vigour found in the rotifer *Brachionus plicatilis*, opportunities for gene flow may occur in small, recently founded populations in which monopolization effects are not yet strong^24^.

The relative contribution and interaction of such evolutionary forces on the genetic makeup of colonising populations is currently much debated. In order to better understand the underlying processes, populations in newly colonised habitats must ideally be tracked over several years. However, evolutionary studies of colonisation in the wild are difficult and labour intensive, and very rarely have natural populations been tracked from the first stages of colonisation, partly due to the fleeting nature of the colonisation process on an ecological scale^18^,^25^,^26^. In this sense, newly created aquatic habitats offer a unique opportunity to investigate the dynamics of zooplankton colonisation and the interplay of evolutionary forces from the earliest stages of population foundation^5^,^18^,^23^. Zooplankton provide excellent models for a cost-effective evaluation of the colonisation success of new habitats, both from an ecological^27^,^28^ and a genetic^23^,^29^ perspective. Zooplankters that disperse passively via diapausing eggs (e.g. monogonont rotifers, cladocerans, calanoid copepods) can colonise habitats from a few propagules (as few as 1 to 3 in *Daphnia*^9^). Diapausing eggs reach new habitats through wind, water flow and animal-mediated transport^30^, and are able to cope with extreme environmental conditions and desiccation, forming extensive dormant egg banks, which remain viable for a long time in the sediments^16^,^31^.

In the present study, we took advantage of the construction of temporary ponds in a restored marshland in one of the most important European wetlands (Doñana National Park, SW Spain)^32^ to track the first stages of zooplankton colonisation. Ponds were arranged in two major blocks of 44 ponds each, plus eight isolated ponds in an estate of former farmland. We analysed the patterns of clonal diversity and genetic structure of newly established populations of the cyclical parthenogenetic rotifer *Brachionus plicatilis*, during the first three hydroperiods after pond construction. According to the Monopolisation hypothesis, and assuming an absence of historical egg banks due to either aging or pond excavation, we aimed at testing the following specific hypotheses: (i) new populations are founded by few diapausing eggs dispersing from external sources, resulting in low clonal richness and no linkage or HW equilibrium for the new populations in the first hydroperiod; (ii) limited or no increase of allelic diversity over subsequent hydroperiods would be observed when persistent strong founder effects prevent the successful establishment of immigrant genotypes; (iii) genetic drift should result in increasing population genetic differentiation with time.

## Results

### *Brachionus plicatilis* populations

Over the course of the three studied hydroperiods, the *B. plicatilis* species complex was found in 36 out of the 58 sampled new ponds and in one of eight reference sites in the surrounding area (Supplementary Table S1 for densities of the species complex over the study period in each pond). Of these 36 ponds, 17 were not studied further as rotifer densities were extremely low over the three hydroperiods (total accumulated density <5 ind·L^−1^). Four populations detected for the first time in the 3^rd^ hydroperiod (4N3, 1S1, 4S3 and 7S3) were not analysed as these ponds had not been sampled in previous hydroperiods (see Supplementary Table S1).

**Figure 1:**
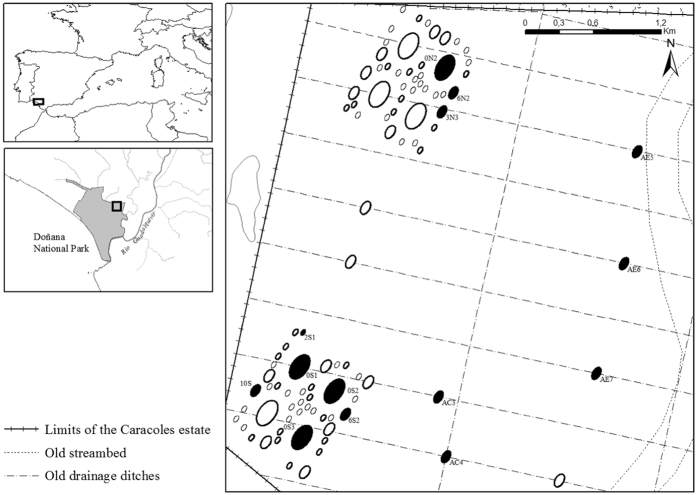
Study area and map of the newly constructed temporary ponds. The subset of new ponds investigated is highlighted with a bold edge. Black filled ponds are those selected for the study of populations of *B. plicatilis sensu stricto*. Drainage ditches present prior to restoration are also shown (hatched straight lines). This map was produced with the online software ArcGIS (https://www.arcgis.com/).

A total of 27 samples from the three hydroperiods with a high proportion of *B. plicatilis sensu stricto* individuals (*B. plicatilis* hereafter), according to positive species-specific *Bp1b* PCR amplification^33^, were selected for genetic analysis. These samples belonged to 14 new ponds: 9 within-block ponds and 5 isolated ponds (Fig. 1). No *B. plicatilis* was found in any of the reference sites (see Supplementary Table S1).

**Figure 2:**
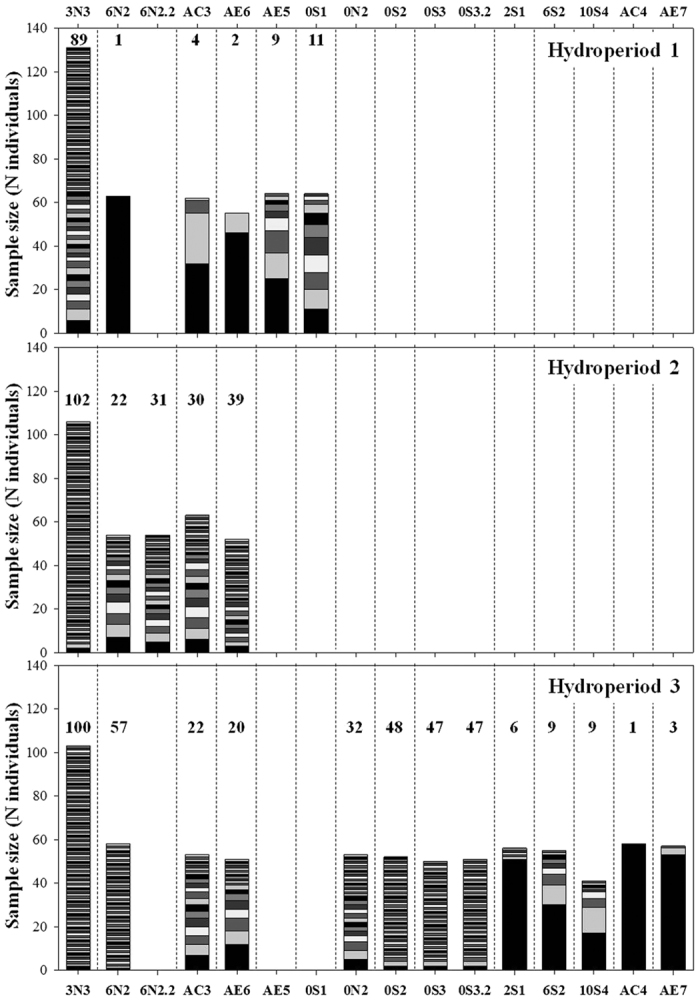
Sample size (y axis) and the estimated number of clones of *B.* *plicatilis sensu stricto* in each sample during three hydroperiods. The name of the new pond to which each sample belongs is shown at the top and the bottom of the plot. Each shade in the stacked bars represents a different clone, but the same shade does not represent the same clone in different samples. The total number of clones found in each sample is given above each stack bar.

Only four of the 14 ponds studied held *B. plicatilis* populations in all three hydroperiods: two ponds in the northern block, 3N3 and 6N2, and two isolated ponds, AC3 and AE6. Populations in ponds 0S1 and AE5 were only analysed in the 1^st^ hydroperiod either because we could not detect them thereafter, or because their densities were too low for analysis (see Fig. 2 and Supplementary Table S1). Eight additional populations were analysed in the 3^rd^ hydroperiod (ponds 0N2, 0S2, 0S3, 2S1, 6S2, 10S4, AC4 and AE7). In the 3^rd^ hydroperiod, populations showed high densities and the species became widespread. We cannot exclude the presence of populations in the southern block of ponds during the 2^nd^ hydroperiod since sampling was prevented during most of the hydroperiod (see Methods).

### Allelic richness

**Figure 3:**
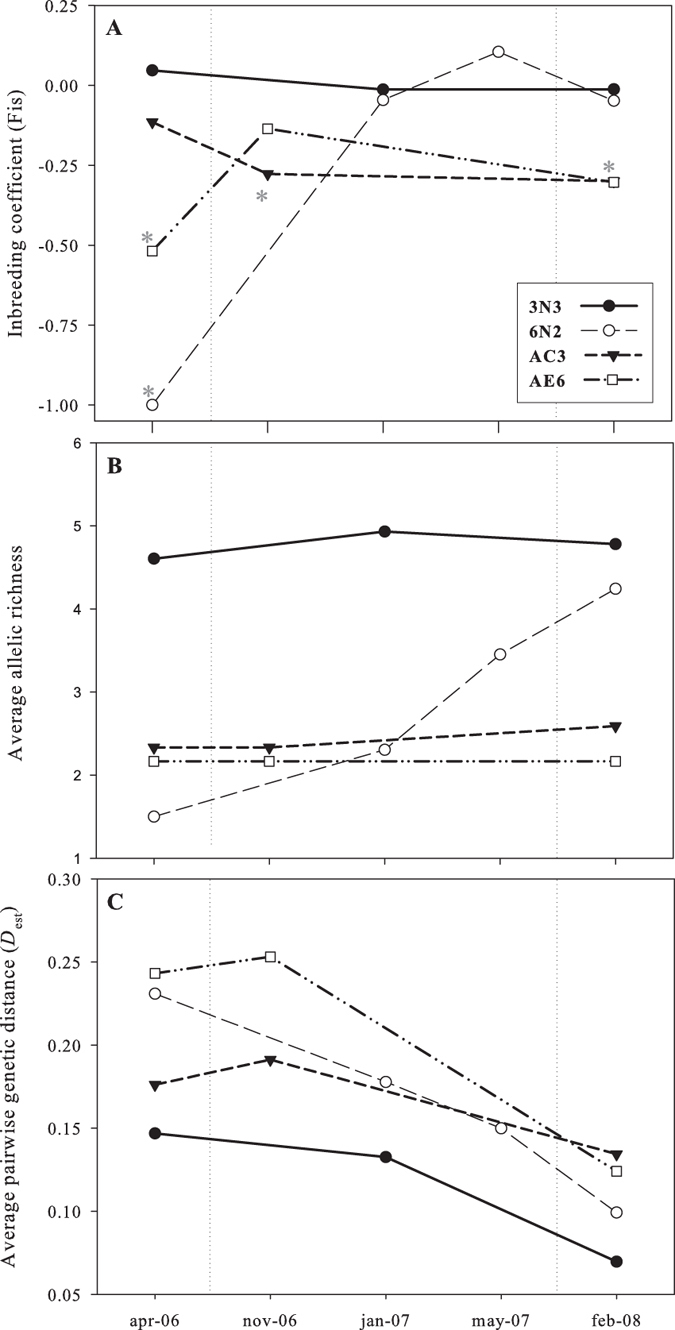
Temporal patterns of genetic parameters from new ponds with *B. plicatilis sensu stricto* populations in all three hydroperiods. (**A**) inbreeding coefficient (F_IS_), (**B**) allelic richness adjusted for minimum sample size, and (**C**) average pairwise genetic differentiation (*D*_est_) among each pond and the others within each hydroperiod. (*) Indicates Fis values significantly different from zero. Note the y axis is not to scale. Vertical dotted lines separate hydroperiods.

Six loci were polymorphic in the populations studied with 6 alleles in *Bp1b*, 6 alleles in *Bp2*, 5 alleles in *Bp3c*, 14 alleles in *Bp4a*, 4 alleles in *Bp5d* and 3 alleles in *Bp6b*, which were used to construct the multi-locus genotype (MLG, also see next section) for each individual. Despite previous successful amplification in a control population (Torreblanca Marsh, Spain)^13^, locus *Bp3* could not be used in our populations due to inconsistent amplification. Due to the low (0.012 overall) frequency of null alleles across microsatellite loci and populations, correction for null alleles was not performed. The least polymorphic locus *Bp6b* was fixed in 14 of the 27 samples for one allele, which in the remaining samples showed a frequency ≥67%. Private alleles were found only in the most polymorphic locus *Bp4a*: (one allele in 6N2, one in 3N3, and one in 10S4 with frequencies of 0.028, 0.010 and 0.012, respectively). The number of polymorphic loci per sample ranged from 3 to 6, and the total number of alleles per sample from 11 to 30. The allelic richness per sample, averaged over loci and adjusted for the minimum sample size (41 individuals), ranged from 1.50 to 4.85 (Table 1). No linkage disequilibrium was found for any of the studied loci in the analysed samples. Figure 3 depicts the temporal patterns in all three hydroperiods of the genetic parameters observed in the new ponds with *B. plicatilis* populations (i.e. (A) inbreeding coefficient (FIS), (B) allelic richness, and (C) average pairwise genetic differentiation (Dest)).

Of the four populations that were present throughout the study period, the number of alleles and allelic richness was highest in population 3N3 and remained stable in all three hydroperiods (29 to 31 alleles, Table 1, Fig. 3B). In population 6N2, the number of alleles increased from 9 (in the single founding clone) to 26 in the 3^rd^ hydroperiod, with its allelic richness increasing from 1.50 to 4.07 (Fig. 3B) indicating successful immigration of new clones (Table 1, see Supplementary Fig. S1). In contrast, in the two isolated populations, the number of alleles and allelic richness either increased only slightly through time (AC3, 14 to 16 alleles) or remained stable (AE6, 13 alleles)(Table 1).

### Multilocus genotypes and HWE

The number of MLGs per sample per hydroperiod ranged from 1 to 102 (Fig. 2 and Table 1). All *B. plicatilis* populations from the 1^st^ hydroperiod, except 3N3, had a low number of MLGs (<12), which could be regarded as an accurate estimate of the number of clones according to GenClone 2.0 (see Methods). In the most extreme case, all individuals of population 6N2 (N = 63) shared the same MLG, and thus this was considered a monoclonal population in the 1^st^ hydroperiod (Fig. 2). In the course of the study period, the number of clones and clonal diversity increased in this population. Five of the populations detected for the first time in the 3^rd^ hydroperiod also had a low number of MLGs (<10) and low clonal diversities (Table 1), suggesting recent colonisation and population establishment.

**Figure 4:**
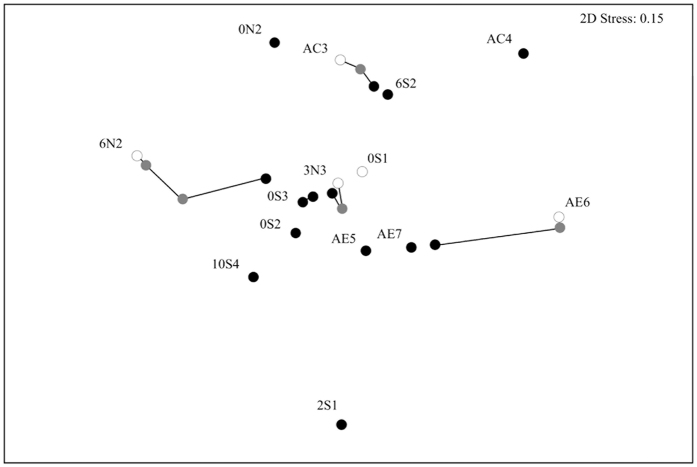
Genetic differentiation (*D*_est_) among *B. plicatilis* populations (NMDS plot). Symbols in different colours separate the hydroperiods: white for the first, grey for the second and black for the third. Lines join samples of the same pond from the three hydroperiods.

Significant heterozygote excess (negative F_IS_, Table 1) was common in ponds with low clonal diversity in their first hydroperiod, an indication that the few heterozygous individuals that colonised the ponds proceeded to reproduce parthenogenetically. Both isolated populations, AC3 and AE6, had a significant excess of heterozygotes (positive F_IS_, Table 1) during almost the entire study period, and their clonal diversity decreased in the 3^rd^ hydroperiod (Table 1). In contrast to the other three populations detected throughout the three hydroperiods, we observed high clonal diversity and genetic stability across hydroperiods in population 3N3 (Figs 1 and 4).

Patterns of HWE differed among the four populations detected throughout the three hydroperiods (Table 1): populations 6N2, AC3 and AE6 departed from HWE in at least one of the studied hydroperiods due to heterozygote excess (Table 1), while population 3N3 was in HWE in all three hydroperiods.

### Patterns of genetic differentiation

**Figure 5:**
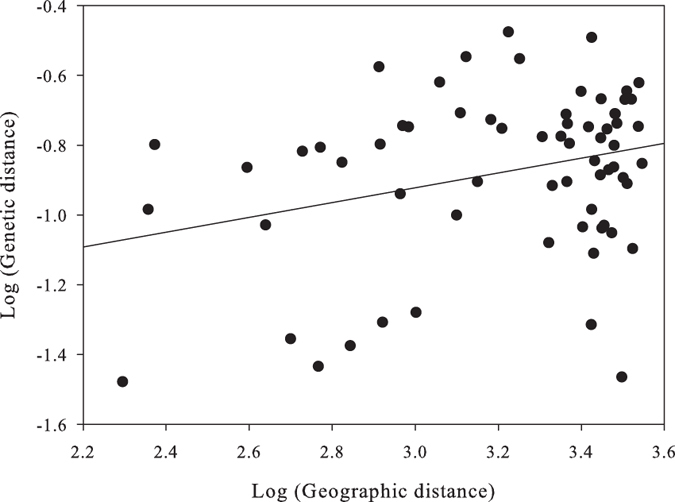
Isolation by distance plot of *D*_est_ versus geographic distance (m) of all pairwise combinations of samples for the third hydroperiod. Note the logarithmic scale for both axes (Mantel R = 0.292, *p*-value = 0.0143).

When considering all populations studied within each of the three hydroperiods, the overall genetic differentiation was very similar in each hydroperiod. It increased slightly from *D*_est_ = 0.161 (*p* = 0.001) in the 1^st^ hydroperiod to *D*_est  _ = 0.183 in the 2^nd^ (*p* = 0.001) and it decreased to *D*_est_ = 0.150 (*p* = 0.001) in the 3^rd^ (Fig. 4). A spatial pattern of genetic differentiation among ponds was also observed in the 3^rd^ hydroperiod (Fig. 5; Mantel test, R = 0.292, *p* = 0.014, 9999 permutations).

The four populations that persisted throughout all three hydroperiods became more similar through time, converging on the highly diverse 3N3. The decrease in the average pairwise genetic differentiation observed between the 2^nd^ and 3^rd^ hydroperiod (Fig. 3C) was mainly driven by populations in which allelic diversity increased (6N2 and AC3), eventually becoming more similar to 3N3 (see NMDS plot in Fig. 4), which suggest the latter as a source population. In between the isolated populations, genetic differentiation was always higher than in the within-block populations. AMOVA results regarding these four populations (see Supplementary Table S2) indicate non-significant, low genetic differentiation among them in the 1^st^ hydroperiod (F_ST_ = 0.019, *p* = 0.23). Although differentiation increased and became significant in the 2^nd^ hydroperiod (F_ST_ = 0.14, *p* < 0.01), it decreased again in the 3^rd^ hydroperiod (F_ST_ = 0.07, *p* < 0.01).

## Discussion

The understanding of the early stages of habitat colonization is essential for a successful restoration of aquatic ecosystems. The construction of new, temporary ponds as part of a marshland restoration project, together with the high dispersal ability of rotifers^34^,^35^ offered a unique opportunity to study early zooplankton colonisation patterns. We explored the genetic diversity and structure of newly established *Brachionus plicatilis* populations throughout the first three hydroperiods of a subset of these ponds. Our results did not indicate a uniform colonisation pattern among the populations, highlighting the complexity of the colonisation process during these initial stages, with evidence for persistent founder effects in some populations, but also successful migration and subsequent homogenisation in other populations.

Over 60% of the new ponds monitored were colonised by the *B. plicatilis* species complex, which rapidly established large populations within three years of pond construction. Six out of the 14 populations analysed were detected during the 1^st^ hydroperiod and attained considerable densities within weeks of the first flooding. Five populations were first detected in the 3^rd^ hydroperiod, indicating that additional ponds continued to be colonised throughout the study period.

In support of the first hypothesis, we found that five of the six populations detected in the 1^st^ hydroperiod had low values of genetic and clonal diversity of one to 11 clones (Table 1), indicating their recent founding by a small number of propagules, as observed in *Daphnia* populations^17^,^18^. In contrast, the high clonal diversity of the sixth population (3N3) and the presence of HW equilibrium suggest colonisation from a pre-existing egg bank which may have persisted from historic water bodies present in the marsh prior to the farmland conversion (see Methods). Zooplankton egg banks are important reservoirs of biological and genetic diversity^16^,^36^,^37^ that can maintain viable eggs during decades (60–80 years in *Brachionus plicatilis*^38^) or even centuries (e.g. ~700 yr in *Daphnia*^39^; >300 yr in calanoid copepods^40^). During pond construction, former agricultural drainage ditches were filled with topsoil from the surrounding area, where diapausing propagules may have persisted. Although a previous study suggested low densities of any remaining historical egg banks^41^, the fact that pond 3N3 partly overlay one of these ditches could explain the observed genetic pattern. In contrast, the high genetic diversity of some populations first detected in the 3^rd^ hydroperiod is more difficult to explain. We may have failed to detect these populations in previous hydroperiods because abundance was below our detection limit or because the area where these ponds were located (southern block) was inaccessible for sampling due to flooding. All these ponds became part of a larger flooded area connecting several ponds in which *B. plicatilis* may have been present but that were not sampled (see Methods).

Given that *B. plicatilis sensu stricto* was not recorded in any of the reference sites, the sources of colonists for populations founded by a small number of clones remain uncertain. However, the lack of significant genetic structure at the onset of colonisation (AMOVA results, see Supplementary Table S2), suggests that ponds were colonised by a closely related subset of clones. Doñana is a large wetland complex containing highly diverse water bodies^42^ and the species complex of *B. plicatilis* has been widely recorded in the area and in other Andalusian wetlands^43^,^44^, including populations of *B. plicatilis sensu stricto*^45^. Moreover, previous results from a community ecology study in the same restored marshland^35^ do not support dispersal limitation for rotifers at such small spatial and short time scales.

We did not find clear evidence in favour or against persistent founder effects as patterns were not consistent with either. A significant population structure from the early colonisation stages due to chance events may be expected, especially if clonal diversity of source populations is high (i.e. as every pond would be colonised by a different set of clones). When persistent founder effects are present, an increase in genetic differentiation between populations over time can be expected^12^. In contrast, in our study no significant genetic structure was found at the onset of colonisation and although differentiation between all studied populations increased in the second hydroperiod, it dropped again to a much lower value in the third hydroperiod (AMOVA results, see Supplementary Table S2). Importantly, this pattern was also observed in the four populations present throughout the study, suggesting that it was unaffected by new populations established in the 2^nd^ and 3^rd^ hydroperiod.

The decrease in genetic differentiation observed in the 3rd hydroperiod was driven by two adjacent populations (3N3 and 6N2), suggesting gene flow. The absence of persistent founder effects in recently founded populations, like 6N2, may result from a small egg bank with little buffering capacity against migration. The size of the egg banks produced during the early stages of colonisation is likely to be smaller than those of mature habitats, and may have made a limited contribution to population dynamics^20^. We did not expect founder effects in 3N3 since this pond was presumably re-established from a pre-existing large egg bank of a historical, mature population with a high genetic and clonal diversity.

The four populations followed throughout three hydroperiods presented diverse patterns: the population presumably re-established from a historical egg bank (3N3) showed high and stable allelic and clonal diversity throughout the study. In the two spatially isolated ponds (AC3 and AE6), which were never connected to other ponds by flooding, allelic diversity was low and increased only slightly over time. The clonal diversity in these ponds increased exclusively by recombination during the sexual phase, given the absence of new alleles (Appendix 3). The significant spatial pattern found in the 3rd hydroperiod may suggest low immigration rates in the isolated populations compared to those in within-block ponds. If the number of immigrant genes is limited, and they do not disappear by drift, their frequencies in populations are expected to be low and to increase very slowly^29^, taking longer to be detected. Apart from low immigration rates, the observed pattern of stable, low allelic richness could be caused either by persistent founder effects, or by a combination of both. Finally, in the population that initially had a single-clone (6N2) we found a steady increase of allelic richness throughout the study period. This population is located at a short distance from the large, genetically diverse 3N3 population within the northern block and higher dispersal rates could be expected. Heterosis, and spread of immigrant genes within the inbred population^18^,^19^,^26^,^46^, may also have contributed to the increase in genetic diversity observed in this pond.

Hardy-Weinberg equilibrium was established after the 1^st^ hydroperiod in population 6N2 that showed a continuous increase of allelic richness and clonal diversity. This population was founded by only one clone, presumably resulting in selfing for the production of dormant eggs at the end of the 1^st^ hydroperiod. This population is likely to have suffered from strong inbreeding depression at the onset of the 2nd hydroperiod, providing a competitive advantage to immigrant genotypes, as observed in *Daphnia* metapopulations^19^,^22^,^23^. In contrast, the isolated populations AC3 and AE6 with stable allelic diversity showed evidence of heterozygote excess maintained over time. This could be due to colonisation by a few heterozygous individuals in the 1^st^ hydroperiod, and strong selection for heterozygous individuals during the following hydroperiods^17^,^47^.

## Conclusions

Overall, our study, although admittedly based on a small number of ponds, revealed that the colonisation of a cyclical parthenogenetic rotifer in newly created habitats was subject to a high level of stochasticity, at least during the early stages of the process. Founder effects were not expected in the population re-established from pre-existing egg banks, but were not apparent in the pond founded by a single clone, which showed that immigration might be important when bottlenecks are very strong. Even in those populations founded by few genotypes, the persistent founder effects were difficult to detect. Spatial isolation of these ponds may mask the founder effects, lowering the immigration rates in comparison to the ponds located within blocks. Whether persistent founder effects may establish during later years remains to be tested, similar to a study by Ortells *et al*.^23^ regarding *Daphnia* colonisation. Our results suggested that most of the colonising propagules came from external sources, but they also highlight the role of historical egg banks, as a local “genetic archive”, in facilitating re-colonisation and the build-up of new populations in restored habitats^16^.

## Methods

### Study species

*Brachionus plicatilis sensu stricto* is a cosmopolitan rotifer that inhabits salt lakes and brackish coastal lagoons^11^. It belongs to a cryptic species complex, with six species present in the Iberian Peninsula, which can only be reliably identified by molecular markers and can co-occur in the same habitat^48^,^49^. The species reproduces by cyclical parthenogenesis: the life cycle begins with the hatching of a sexually-produced, genetically unique diapausing egg in response to hatching cues. Massive hatching is thought to occur more or less synchronously in a population, mainly in the early stages of pond refilling, allowing this species to quickly reach high densities in the habitat. Hatchlings are diploid, parthenogenetic (amictic) females that produce genetically identical offspring (clones). Parthenogenetic or clonal reproduction occurs for an indefinite number of generations as long as conditions are favourable (parthenogenetic phase). Sexual reproduction takes place when external stimuli (e.g. population density, photoperiod) induce some females to produce sexual (mictic) daughters, which produce haploid sexual eggs. If unfertilized, these eggs develop to haploid males; otherwise they produce diploid diapausing eggs that accumulate in the sediment until external stimuli (e.g. light and oxygen) induce hatching.

### Study site

The studied ponds are located within Caracoles estate, in Doñana National Park (SW Spain, Fig. 1), a former seasonally flooded marshland area of 27 Km^2^ turned into arable farmland in the 1960 s after drainage. Between summer 2004 and spring 2005, 96 experimental ponds were dug in the estate as part of a restoration project, ‘The Doñana 2005 restoration plan’^50^. Ponds were arranged in two major blocks of 44 pools each, plus eight spatially isolated ponds distributed throughout the estate. Some of them partly overlay former drainage ditches previously filled with topsoil from surrounding areas (Fig. 1). The ponds fill primarily by rainfall and local surface run-off and are frequented by a diverse waterbird community^32^. During and after major rainfall events, some ponds temporarily overflow and connect to flooded grassland areas and/or to neighbouring ponds. All ponds dry out completely during the summer, even in the wettest years.

### Sample collection

A subset of initially 48 new ponds representative in terms of size, depth and connectivity was selected for sampling, including ponds from both major blocks and isolated ponds (Fig. 1). Eight nearby natural and semi-natural temporary water bodies, where *B. plicatilis* species complex had been previously detected^34^,^44^, were selected as reference sites (source populations) to be sampled simultaneously (for details see refs 34,35). Sampling was carried out bimonthly during the first three hydroperiods (occasionally monthly for some ponds; see Supplementary Table S1). In the last hydroperiod, 10 additional ponds were included in the study making a total of 58 new ponds.

The autumn/winter following pond construction was exceptionally dry, and the first flooding event for all new ponds occurred in January 2006. The duration of the first hydroperiod varied among ponds, with some beginning to dry out in early May and others persisting until late June 2006. In the 2^nd^ hydroperiod that started in late October 2006, the first ponds dried out in early May and some persisted until July 2007. During the wettest period, in winter 2007, the southern block of ponds was inaccessible due to flooding of access routes. The 3^rd^ hydroperiod started in December 2007 and lasted until late June 2008. This was the driest hydroperiod and only a small number of ponds, mainly in the northern block, could be sampled (see Supplementary Table S1).

Two zooplankton samples were collected during each pond visit: one for taxonomic identification and counting (preserved in 70% ethanol or lugol), and another for genetic analysis (preserved in absolute ethanol). No genetic samples were available in May 2006. Samples were kept in the dark at 4 °C until DNA was extracted. Each sample was obtained by filtering a minimum of 20 L of water through a 64 μm net. To avoid dispersing zooplankton and sediment transfer among ponds, the sampling equipment was rinsed with tap water and 70% ethanol between ponds, and boots were covered with plastic bags (see ref. 34 for details on zooplankton sampling and processing).

### Microsatellite genotyping and analyses

For samples containing individuals of the *Brachionus plicatilis* species complex, we used positive amplification of the species-specific *Bp1b* microsatellite locus^33^ to identify individuals of *B. plicatilis sensu stricto*. For this purpose, a total of 916 individuals (minimum sample size of 19) were screened (see Supplementary Table S1). DNA was extracted from individual females using a modified HotSHOT protocol^51^, with a volume of 25–25 μL for lysis and neutralization solutions. PCR amplifications were performed in a reaction volume of 10 μL containing 2 μL of template DNA, 1 × NH_4_ PCR buffer (BIOLINE), 0.2 mM dNTPs, 0.5 μM of each primer, 1.5 mM MgCl_2_ and 0.025 U of *Taq* DNA polymerase (BIOLINE). PCR reactions were performed in a Veriti^®^ Fast Thermal Cycler (Applied Biosystems) as described in ref. 33. Three μl of the PCR products were separated by electrophoresis in a 2% agarose gel in 1x TBE buffer and stained with ethidium bromide for band visualization. For logistic reasons, only those samples with a large proportion of *B. plicatilis* individuals were selected for further study.

Between 41 and 66 individuals per sample (depending on availability) were genotyped for seven trinucleotide microsatellite loci (*Bp1b, Bp2, Bp3, Bp3c, Bp4a, Bp5d, Bp6b*)^33^. All loci were amplified in a single multiplex PCR following a protocol optimized for the present study. Multiplex PCRs were performed in a reaction volume of 10 μL containing 2 μL of template DNA, 5 μL of 2x QIAGEN^®^ Multiplex PCR Master Mix (including 3 mM MgCl_2_, dNTP Mix and HotStarTaq^®^ Polymerase) and 2 μL of RNAse-free water. Each reverse primer was labelled in 5′ with a fluorescent dye (Cy5, Cy5.5, MWG Biotech; WellRED D2-PA, Sigma-Aldrich) for their detection in capillary electrophoresis. Primer concentration was optimized to improve amplification performance given the different strength of the fluorescent dyes to 0.1 μM for the Cy5-labelled *Bp4a* and *Bp6b*, 0.2 μM for the Cy5.5-labelled *Bp2, Bp3c* and *Bp5d*, 0.3 μM for D2-PA-labelled *Bp1b* and 0.4 μM for Cy5-*Bp3*. The multiplex PCR programme used was following QIAGEN recommendations. Diluted PCR products (1:20) were combined with a 400 bp-size standard and separated on a Beckman-Coulter CEQ^TM^ 8000. Alleles were scored using the CEQ Fragment Analysis software (Beckman-Coulter^TM^) and then checked manually.

A multi-locus genotype (MLG) was constructed for each individual based on its genotype at each individual locus to infer the clonal structure of the populations^21^. Within each sample, those individuals with the same MLG are likely to belong to the same clone (i.e. offspring of a genetically unique female). The program GenClone 2.0^52^ was used to test if the number of loci and the sample size used were sufficient to accurately estimate the number of clones present in each sample, or otherwise, whether it should be considered an underestimate. A measure of clonal diversity (D*, Simpson complement), which combines richness and evenness^52^ was also obtained using this program.

The frequency of null alleles and pairwise F_ST_ values were calculated with FreeNA^53^. Other parameters of genetic diversity such as the number of polymorphic loci (PL), the total number of alleles (A) and the average expected heterozygosity at Hardy–Weinberg equilibrium (He) were obtained using GenAlEx 6.4^54^. FSTAT 2.9.3^55^ was used to estimate allelic richness (Rs), as the average number of alleles per locus adjusted for the minimum sample size (41 individuals), using a rarefaction method^56^, as well as the inbreeding coefficient (Fis) over loci within each population testing for significant deviations from Hardy-Weinberg equilibrium (based on 2880 randomizations). Linkage disequilibrium tests between all pairs of loci (based on 7200 permutations) were also calculated.

To test for temporal changes in the amount of genetic variance between and among populations, we performed analyses of molecular variance (AMOVA) based on *F*_ST_ in each hydroperiod separately, using GenAlEx 6.4^54^. We performed AMOVA on a subset of data that contained only the populations analyzed throughout three hydroperiods, using only one individual per MLG to avoid the bias introduced by clonal reproduction. Genetic differentiation between populations was estimated using the unbiased differentiation statistic *D*_est_^57^. The R-package DEMEtics^58^ was used to calculate *D*_est_ values, 95% confidence intervals and *p*-values (null hypothesis of zero differentiation) by means of 1000 bootstrapping iterations. All *p*-values were adjusted using Bonferroni correction for multiple tests. Pairwise measures of *D*_est_ were used as a distance matrix in a nonmetric multidimensional scaling ordination (NMDS) to provide a visual representation of the genetic variability among populations. Isolation by distance was assessed by Mantel test (9999 permutations), testing for relationships between genetic and geographic distances.

## Additional Information

**How to cite this article:** Badosa, A. *et al*. Isolation mediates persistent founder effects on zooplankton colonization in new temporary ponds. *Sci. Rep.* **7**, 43983; doi: 10.1038/srep43983 (2017).

**Publisher's note:** Springer Nature remains neutral with regard to jurisdictional claims in published maps and institutional affiliations.

## Supplementary Material

Supplementary Information

## Figures and Tables

**Table 1:** Genetic diversity measures of the *Brachionus plicatilis sensu stricto* populations.

POND	Hydroperiod		Parthenogenetic phase	N	MLG	D*	PL	A	Rs	He	F_IS_
3N3	1	Mar + Apr-06	1^st^	129	89	0.99	6	30	4.50	0.444	0.05
	2	Jan + Mar-07	2^nd^	104	102	1.00	6	31	4.85	0.494	−0.01
	3	Feb + Apr-08	3^rd^	101	100	1.00	6	29	4.73	0.490	−0.01
6N2	1	Apr-06	1^st^	63	1^●^	0.00	3	9	1.50	0.250	−1.00*
	2	Jan-07	2^nd^	54	22	0.95	3	14	2.19	0.260	−0.05
	2	May-07	2^nd^	54	31	0.98	5	21	3.28	0.335	0.11
	3	Feb-08	3^rd^	57	57	1.00	4	26	4.07	0.451	−0.05
AC3	1	Apr-06	1^st^	62	4^●^	0.60	4	14	2.33	0.292	−0.12
	2	Nov-06	2^nd^	63	30	0.96	4	14	2.33	0.288	−0.28*
	3	Feb-08	3^rd^	53	22	0.95	6	16	2.59	0.310	−0.30*
AE6	1	Apr-06	1^st^	55	2^●^	0.28	4	13	2.17	0.384	−0.52*
	2	Nov-06	2^nd^	51	39	0.99	4	13	2.17	0.397	−0.14
	3	Feb-08	3^rd^	51	20	0.91	4	13	2.17	0.369	−0.30*
AE5	1	Apr-06	1^st^	64	9^●^	0.78	6	23	3.70	0.364	−0.02
0S1	1	Apr-06	1^st^	64	11^●^	0.90	5	24	3.82	0.418	0.01
0N2	3	Feb-08	?	53	32	0.98	6	24	3.76	0.357	−0.22*
0S2	3	Dec-07	?	51	48	1.00	6	21	3.48	0.392	−0.03
0S3	3	Dec-07	?	49	47	1.00	6	24	3.89	0.400	−0.05
	3	Apr-08	?	50	47	1.00	6	21	3.40	0.379	−0.03
2S1	3	Apr-08	1^st^	56	6	0.17	5	17	2.67	0.287	−0.74*
6S2	3	Apr-08	1^st^	55	9	0.67	4	17	2.77	0.326	−0.08
10S4	3	Feb-08	1^st^	41	9	0.74	5	19	3.17	0.343	−0.31*
AC4	3	Apr-08	1^st^	58	1^●^	0.00	5	11	1.83	0.417	−1.00*
AE7	3	Apr-08	1^st^	57	3	0.13	4	13	2.02	0.260	−0.93*

N, sample size; MLG, number of clones; D^*^, clonal diversity; PL, number of polymorphic loci; A, number of alleles; Rs, average allelic richness corrected for the sample size; He, average expected heterozygosity; Fis, inbreeding coefficient where (*) indicates significance deviation from HWE at *p*-value < 0.00035 after Bonferroni correction. The MLG estimates with (^●^) should be considered as the real number of clones according to GenClone 2.0; the rest as underestimates.
