# Supplementary material for: Isolation mediates persistent founder effects on zooplankton colonisation in new temporary ponds
Source: Sci Rep. 2017 Mar 9;7:43983. doi: 10.1038/srep43983 (PMC5343421; doi:10.1038/srep43983)

Supplementary Information for

**Isolation mediates persistent founder effects on zooplankton colonisation in new temporary ponds.**

Anna Badosa^1,2,5^, Dagmar Frisch^3^, Andy J. Green^1^, Ciro Rico^1,4^ & Africa Gómez^5^

**Affiliations**

^1^ Department of Wetland Ecology, Doñana Biological Station (EBD-CSIC), c/Américo Vespucio s/n, 41092 Sevilla, Spain.

^2^ Aquatic Ecology Group, BETA Technology Centre, University of Vic - Central University of Catalonia, c/ de la Laura, 13, 08500 Vic, Spain

^3^ School of Biosciences, University of Birmingham, B15 2TT, United Kingdom

^4^ School of Marine Studies, Molecular Diagnostics Laboratory, The University of the South Pacific, Laucala Campus, Suva, Fiji

^5^ School of Environmental Sciences, University of Hull, HU6 7RX, United Kingdom

Corresponding author:

Professor Ciro Rico email: ciro.rico@usp.ac.fj

**This Supplementary Information includes:**

Supplementary Tables 1 and 2

Supplementary Figure 1

**Supplementary Table 1.** Density (ind·L^-1^) of *Brachionus plicatilis* species complex in the new ponds during the first three hydroperiods. Grey columns indicate the dry seasons where all ponds dried out. Zero density values indicate the absence of the species complex in the zooplankton samples whereas (n.s.) indicates those months where sampling was not performed. Last column shows the total accumulated density throughout the three hydroperiods. Superscripts indicate the number of individuals with positive amplification of the species-specific *Bp1b* microsatellite (i.e. *B. plicatilis* sensu scripto) with respect to the total number screened (see methods). Samples selected for genetic analysis are framed in black, and the ponds they came from are highlighted in bold. (^D^) indicates those ponds that were built on former drainage ditches.

|  | | **HYDROPERIOD 1** | | | | |  | |  | | **HYDROPERIOD 2** | | | |  | | **HYDROPERIOD 3** | | | |  |  | |  | |
| --- | --- | --- | --- | --- | --- | --- | --- | --- | --- | --- | --- | --- | --- | --- | --- | --- | --- | --- | --- | --- | --- | --- | --- | --- | --- |
| **SAMPLING POINTS** | | **FEB 06 MAR 06 APR 06MAY 06** | | | | |  | | **NOV 06 JAN 07 MAR 07 APR 07 MAY 07** | | | | | |  | | **DEC 07** | | **FEB 08** | | **APR 08** |  | | **TOTAL** | |
| **NORTH-BLOCK PONDS:** | |  | | | | |  | |  | |  | | | |  | |  | |  | |  |  | |  | |
| 0N1 ^D^ | | 0 | n.s. | 1.8 | n.s. | | 0 | | 0 | | n.s. | 0 | n.s. | | almost dry | | | almost dry | almost dry | | | 1.80 | |  |  |
| **0N2** | | **0** | n.s. | **0** | **n.s.** | | **0.05** | | **0** | | **n.s.** | **0** | **n.s.** | | **dry** | | | **_1140.8_ ^63/114^** | **almost dry** | | | **1,140.85** | |  |  |
| 0N3 ^D^ | | 0 | n.s. | 0.05 | n.s. | | 0 | | 0 | | n.s. | 0 | almost dry | | almost dry | | | almost dry | almost dry | | | 0.05 | |  |  |
| 1N2 | | 0 | 0 | 0 | almost dry | | 0.05 | | 0 | | 0 | 0 | almost dry | | dry | | | almost dry | dry | | | 0.05 | |  |  |
| **3N3 ^D^** | | **0** | **_73.33_ ^17/20^** | **503.33 ^19/20^** | **0** | | **0.4** | | **_48_ ^19/20^** | | **_65_ ^19/20^** | **0** | **0** | | **too shallow** | | | **_256_ ^17/22^** | **_6.4_ ^19/20^** | | | **952.46** | |  |  |
| 4N1 | | 0 | 0 | 0.05 | 1055.67 | | 78 | | 0 | | 0 | 0 | 0 | | dry | | | 2468 ^7/22^ | almost dry | | | **3,601.72** | |  |  |
| 4N3 | | n.s. | n.s. | n.s. | n.s. | | n.s. | | n.s. | | n.s. | n.s. | n.s. | | too shallow | | | _18.2_ 11/20 | _2.7_ 14/20 | | | 20.90 | |  |  |
| 4N4 | | 0 | n.s. | 0.05 | dry | | 0 | | 0 | | n.s. | 0 | almost dry | | dry | | | too shallow | dry | | | 0.05 | |  |  |
| 5N2 | | 0 | n.s. | 0.15 | almost dry | | 1 | | 0 | | n.s. | 0 | n.s. | | dry | | | too shallow | dry | | | 1.15 | |  |  |
| **6N2** | | **0** | **0** | **166.67 ^19/20^** | **1411** | | **0.65** | | **_23_ ^19/20^** | | **0.25** | **3.9** | **_9_ ^18/20^** | | **too shallow** | | | **4204.8 ^84/116^** | **too shallow** | | | **5,819.27** | |  |  |
| 9N3 ^D^ | | 0 | n.s. | 0 | n.s. | | 0 | | 0 | | n.s. | 0 | n.s. | | too shallow | | | 0.15 | too shallow | | | 0.15 | |  |  |
| 9N4 ^D^ | | 0 | 0 | 0 | dry | | 0 | | 0 | | 0.25 | 0 | dry | | dry | | | almost dry | dry | | | 0.25 | |  |  |
| **SOUTH-BLOCK PONDS:** | |  |  |  |  | |  | |  | |  |  |  | |  | |  |  |  | | |  | |  |  |
| **0S1 ^D^** | | **0** | **n.s.** | **111.33 ^17/20^** | **n.s.** | | n.s. | | n.s. | | n.s. | **0** | **n.s.** | | **2.8** | | | **0.2** | **0** | | | **114.33** | |  |  |
| **0S2** | | **0** | **n.s.** | **0** | **n.s.** | | n.s. | | n.s. | | n.s. | **0** | **n.s.** | | **14.67** | | **^19/20^** | **4.8** | **0.1** | | | **19.57** | |  |  |
| **0S3 ^D^** | | **0** | **n.s.** | **0** | **almost dry** | | n.s. | | n.s. | | n.s. | **0** | **almost dry** | | **32.53** | | **^19/22^** | **0** | **_272_ ^19/20^** | | | **304.53** | |  |  |
| 0S4 ^D^ | | 0 | n.s. | 0.05 | n.s. | | n.s. | | n.s. | | n.s. | 0 | n.s. | | too shallow | | | 0 | 0.2 | | | 0.25 | |  |  |
| 1S1 | | n.s. | n.s. | n.s. | n.s. | | n.s. | | n.s. | | n.s. | n.s. | almost dry | | 0 | |  | 0.2 | 626.8 ^19/20^ | | | 627.00 | |  |  |
| **2S1** | | **0** | **n.s.** | **_388_ ^0/20^** | **n.s.** | | n.s. | | n.s. | | n.s. | **0** | **almost dry** | | **0** | |  | **0.8** | **_30.8_ ^19/20^** | | | **31.60** | |  |  |
| 3S1 ^D^ | | 0 | 0.1 | 0.05 | n.s. | | n.s. | | n.s. | | n.s. | 0 | 0 | | 0 | |  | 0 | 0 | | | 0.15 | |  |  |
| 3S3 ^D^ | | 0 | 0 | 0 | 0 | | n.s. | | n.s. | | n.s. | 0 | 0 | | 0 | |  | 0 | 1.2 | | | 1.20 | |  |  |
| 4S2 ^D^ | | 0 | 0 | 0.05 | n.s. | | n.s. | | n.s. | | n.s. | 0 | dry | | dry | | | almost dry | dry | | | 0.05 | |  |  |
| 4S3 | | n.s. | n.s. | n.s. | n.s. | | n.s. | | n.s. | | n.s. | n.s. | n.s. | | 0 | |  | _65.2_ 15/22 | 0.3 | | | **65.50** | |  |  |
| 5S4 | | 0 | 0 | 0 | 0.3 | | n.s. | | n.s. | | n.s. | 0 | dry | | 0 | |  | 0 | 0 | | | 0.30 | |  |  |
| **6S2** | | **0** | **0** | **0** | **0** | | n.s. | | n.s. | | n.s. | **0** | **0** | | **0** | |  | **0** | **_167.4_ ^19/20^** | | | **167.40** | |  |  |
| 7S3 | | n.s. | n.s. | n.s. | n.s. | | n.s. | | n.s. | | n.s. | n.s. | n.s. | | 13.17 | | | 39.8 | 0.6 | | | 53.57 | |  |  |
| 9S1 | | 0 | n.s. | 0.15 | dry | | n.s. | | n.s. | | n.s. | 0 | almost dry | | 0 | |  | 0 | 0 | | | 0.15 | |  |  |
| 9S2 | | 0.25 | n.s. | 0 | n.s. | | n.s. | | n.s. | | n.s. | 0 | n.s. | | 0 | |  | 0 | 0 | | | 0.25 | |  |  |
| **10S4** | | **0** | **0** | **0.1** | **0** | | n.s. | | n.s. | | n.s. | **0** | **0** | | **0** | |  | **_5.5_ 20/20** | **0** | | | **5.60** | |  |  |

| **ISOLATED PONDS:** |  |  |  |  |  | |  |  |  |  |  |  |  |  |  |  |  |  |  |  |  |
| --- | --- | --- | --- | --- | --- | --- | --- | --- | --- | --- | --- | --- | --- | --- | --- | --- | --- | --- | --- | --- | --- |
| **AC3 ^D^** | **0** | **7.25** | **46 ^16/20^** | **almost dry** |  |  | | **11.85** | **^18/19^** | **0** | **0** | **0** | **0** |  | **dry** | **5 ^20/20^** |  | **1091.6 ^12/20^** |  | **1,161.70** | |
| **AC4 ^D^** | **0** | **0** | **0.05** | **0.1** |  | | | **0** |  | **0** | **0** | **0** | **almost dry** |  | **0** | **0.55** | **453.85 ^18/20^** | |  | **454.55** | |
| **AE5 ^D^** | **0** | **0** | **_54.67_ ^18/20^** | **434.67** |  | **0** | | |  | **0** | **0** | **0** | **0** |  | **dry** | **dry** |  | **dry** |  | **489.34** | |
| **AE6 ^D^** | **0** | **0** | **_4.45_ ^19/20^** | **almost dry** |  |  |  | **24^18/19^** | | **0** | **0** | **0** | **0.55** |  | **dry** | **14.9 ^18/20^** | **0** | |  | **43.90** | |
| **AE7 ^D^** | **0** | **0** | **0** | **0.15** |  | **0** | | |  | **0** | **0** | **0** | **29.2 ^0/20^** |  | **0** | **0** | **_22.85_ ^18/20^** | |  | **52.20** | |
| AE8 ^D^ | 0 | 0 | 0 | 0 |  | 0 | | |  | 0 | 0 | 0 | 2 |  | 0 | 0 | 0 | |  | 2.00 | |
| AO1 ^D^ | 0 | 0.05 | 0.3 | 2.3 |  | |  | 0 |  | 0 | 0 | 0.15 | almost dry |  | almost dry | 0 | 0 | |  |  | 2.80 |
| AO2 ^D^ | 0 | 0 | 0.05 | 0.65 |  | | | 0 |  | 0 | 0 | 0 | almost dry |  | almost dry | dry |  | dry |  | | 0.70 |
| **REFERENCE SITE:** |  |  |  |  |  | |  |  |  |  |  |  |  |  |  |  |  |  |  |  |  |
|  |  |  |  |  |  |  |  |  |  |  |  |  |  |  |  |  |  |  |  |  |  |
| ENT 1 | 1.2 | n.s. | 0 |  |  | | | 1 |  | 0 | 0.7 | 254.2 ^0/20^ |  |  | 0 | 0 |  | n.s. |  | | 257.10 |

3

**Supplementary Table 2.** AMOVA results for the *B. plicatilis* populations: (A) including all the populations present in each hydroperiod (note that the number of populations is different), and (B) only considering the four populations that were present throughout the three hydroperiods. In the second hydroperiod, only these four populations were detected and, therefore, only one AMOVA result is shown.

A) B)

| **Hydroperiod 1** | 6 populations | |  |  |  | **Hydroperiod 1** | 4 populations | |  |  |  |
| --- | --- | --- | --- | --- | --- | --- | --- | --- | --- | --- | --- |
| **Source** | **df** | **SS** | **MS** | **Est. Var.** | **%** | **Source** | **df** | **SS** | **MS** | **Est. Var.** | **%** |
| **Among Pops** | 5 | 10.082 | 2.016 | 0.035 | 2 | **Among Pops** | 3 | 5.005 | 1.668 | 0.027 | 2 |
| **Within Pops** | 226 | 312.427 | 1.382 | 1.382 | 98 | **Within Pops** | 190 | 265.701 | 1.398 | 1.398 | 98 |
| **Total** | 231 | 322.509 |  | 1.417 | 100 | **Total** | 193 | 270.706 |  | 1.425 | 100 |
|  |  | **P** |  |  |  |  |  | **P** |  |  |  |
| **Fst** | 0.024 | 0.230 |  |  |  | **Fst** | 0.019 | 0.230 |  |  |  |
|  |  |  |  |  |  | **Hydroperiod 2** 4 populations (2 samples from 6N2) | | | | |  |
|  |  |  |  |  |  | **Source** | **df** | **SS** | **MS** | **Est. Var.** | **%** |
|  |  |  |  |  |  | **Among Pops** | 4 | 68.087 | 17.022 | 0.197 | 14 |
|  |  |  |  |  |  | **Within Pops** | 443 | 554.620 | 1.252 | 1.252 | 86 |
|  |  |  |  |  |  | **Total** | 447 | 622.708 |  | 1.449 | 100 |
|  |  |  |  |  |  |  |  | **P** |  |  |  |
|  |  |  |  |  |  | **Fst** | 0.136 | 0.010 |  |  |  |
| **Hydroperiod 3** | 13 populations | |  |  |  | **Hydroperiod 3** | 4 populations | |  |  |  |
| **Source** | **df** | **SS** | **MS** | **Est. Var.** | **%** | **Source** | **df** | **SS** | **MS** | **Est. Var.** | **%** |
| **Among Pops** | 12 | 90.439 | 7.537 | 0.108 | 8 | **Among Pops** | 3 | 31.039 | 10.346 | 0.105 | 7 |
| **Within Pops** | 791 | 1013.252 | 1.281 | 1.281 | 92 | **Within Pops** | 394 | 540.712 | 1.372 | 1.372 | 93 |
| **Total** | 803 | 1103.690 |  | 1.389 | 100 | **Total** | 397 | 571.751 |  | 1.478 | 100 |
|  |  | **P** |  |  |  |  |  | **P** |  |  |  |
| **Fst** | 0.078 | 0.010 |  |  |  | **Fst** | 0.071 | 0.010 |  |  |  |
|  |  |  |  |  |  |  |  |  |  |  |  |

**Supplementary Figure 1.** Allele frequencies of 6 polymorphic microsatellite markers in the experimental ponds during three hydroperiods. Populations that were present in more than one hydroperiod or sampled on more than one date are marked by a box around multiple dates.


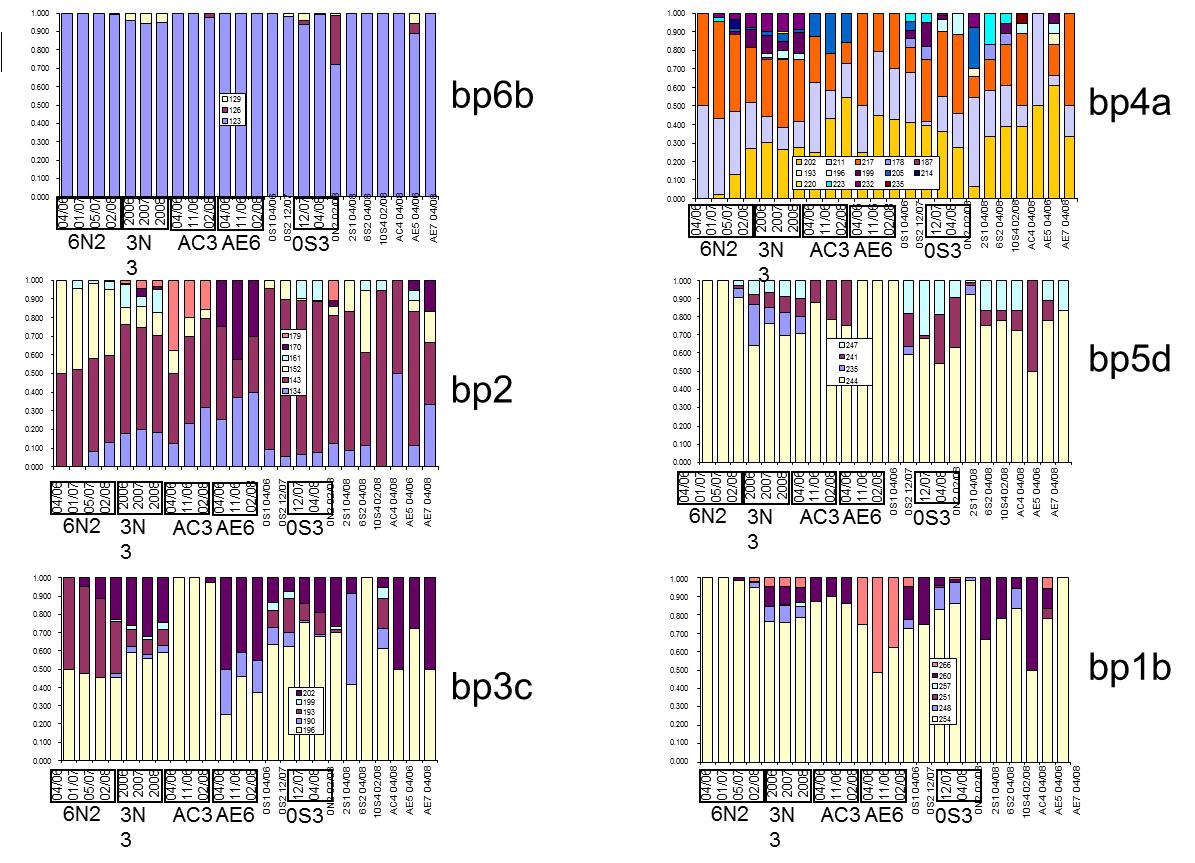

Supplement: Supplementary Information [file srep43983-s1.docx]
